# Salvage circumferential endoscopic submucosal dissection of multifocal Barrett’s adenocarcinoma at an esophagogastric anastomosis

**DOI:** 10.1055/a-2351-2748

**Published:** 2024-07-15

**Authors:** Sunil Gupta, Tony He, Jeffrey D. Mosko, Gary May, Christopher Teshima

**Affiliations:** 1 10071 Therapeutic Endoscopy, St. Michaelʼs Hospital, Toronto, Canada; 2 8539 Gastroenterology and Hepatology, Westmead Hospital, Westmead, Australia

A 69-year-old man underwent neoadjuvant chemoradiotherapy and Ivor Lewis esophagectomy with proximal gastrectomy for T3N0M0 esophageal adenocarcinoma. After 6 years, a new positron emission tomography (PET)-avid thickening was detected at the esophagogastric anastomosis. Endoscopy revealed a 3-cm circumferential segment of Barrett’s mucosa extending from the esophagogastric anastomosis into the remnant esophagus. This contained multiple areas of nodularity with irregular pit and vascular pattern that were concerning for multifocal early Barrett’s cancer ( Fig. 1 ). As the patient was not a candidate for further surgery or radiotherapy, we proceeded with salvage circumferential endoscopic submucosal dissection (ESD) ( Video 1 ).

**Fig. 1:**
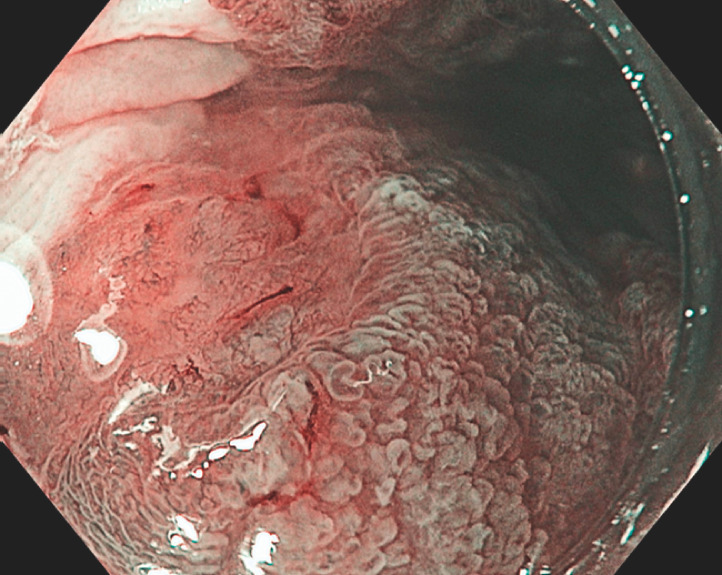
Endoscopic image showing multifocal early Barrett’s adenocarcinoma at the esophagogastric anastomosis.

Salvage circumferential endoscopic submucosal dissection of multifocal Barrett’s adenocarcinoma at the esophagogastric anastomosis in a patient who was not a candidate for further surgery.Video 1

After the distal margin had been marked along the anastomotic line, a circumferential mucosal incision was created ( Fig. 2 **a** ). Submucosal dissection was then performed, aiming to remain above the surgical staples. Following this, four submucosal tunnels orientated at 12, 3, 6, and 9 o’clock were formed from the proximal to the distal margin. Clip-and-snare traction was used by grasping the 12- and 6-o’clock tunnels together ( Fig. 2 **b** ). While this facilitated straightforward dissection of the bridging submucosa between the 6- to 9-, 9- to 12-, and 12- to 3-o’clock tunnels, surgical staples embedded within severe fibrosis were encountered between the 3- and 6-o’clock tunnels ( Fig. 2 **c** ). As this indicated curvilinear proximal extension of the anastomotic line, we aimed to remain below the staples to avoid creating a full-thickness perforation. En bloc resection was achieved with traction assistance and with an occasional underwater approach. An area of muscle injury (10 mm) was closed with through-the-scope clips.

**Fig. 2:**
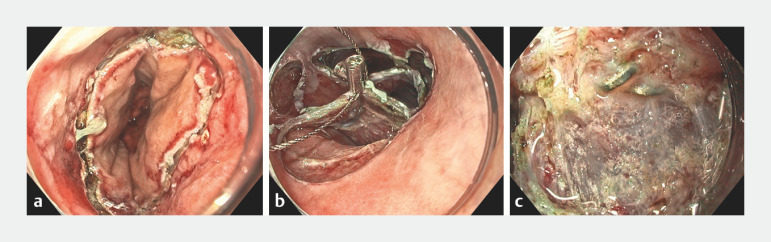
Endoscopic images showing: **a** the distal mucosal incision; **b** clip-and-snare traction being used following the creation of four submucosal tunnels; **c** surgical staples embedded within fibrosis in one of the tunnels.

The final histology revealed R0 excision of a poorly differentiated T1a esophageal adenocarcinoma. Endoscopy at 4 weeks demonstrated healing, with no residual Barrett’s mucosa noted. The patient will be closely monitored with serial imaging, endoscopy, and endoscopic ultrasound examinations 1 .

In nonoperative candidates, salvage ESD of an anastomotic lesion can be a safe and feasible procedure in expert hands. Traction and an underwater approach may assist when encountering areas of fibrosis or surgical staples. Further studies highlighting long-term patient and oncologic outcomes are required.

Endoscopy_UCTN_Code_TTT_1AO_2AG_3AD

## Notes

Endoscopy E-Videos https://eref.thieme.de/e-videos
*E-Videos* is an open access online section of the journal *Endoscopy* , reporting on interesting cases and new techniques in gastroenterological endoscopy. All papers include a high-quality video and are published with a Creative Commons CC-BY license. Endoscopy E-Videos qualify for HINARI discounts and waivers and eligibility is automatically checked during the submission process. We grant 100% waivers to articles whose corresponding authors are based in Group A countries and 50% waivers to those who are based in Group B countries as classified by Research4Life (see: https://www.research4life.org/access/eligibility/ ). This section has its own submission website at https://mc.manuscriptcentral.com/e-videos .
